# Wills’ Hospital

**Published:** 1843-02-04

**Authors:** George H. Burwell

**Affiliations:** Resident Physician


### WILLS' HOSPITAL.

### CLINIC OF DR. ISAAC PARRISH.

[Reported by George H. Burwell, Resident Physician.]

*Wednesday, Jan.* 11, 1843.

Sarah Porter, aged about 20 years, presented herself for advice on account of an encysted tumour of the upper eyelid, near the external canthus—of two years standing.

The tumour was moveable under the skin; and on everting the lid, its base could be distinctly defined, adhering to the tarsal cartilage. It had been for a long time stationary, and caused no pain; but the patient was exceedingly anxious for its removal, on account of the deformity and inconvenience which it produced.

Dr. Parrish remarked, that these morbid growths were very common, particularly in young persons of a strumous constitution, several sometimes occurring at one time, in the same individual, and causing no little uneasiness to anxious friends. They were, however, innocent in their character, and frequently disappeared spontaneously, or under the influence of a mild alterative course, and without any local application. It was better, therefore, not to be in haste about removing them, especially in young persons, but rather to rely upon constitutional means to correct the morbid condition of the system under which they originate. If, however, they have persisted for a long period, it may become necessary to remove them.

There are two methods whereby this may be accomplished. First, by external incision through the integuments and orbicularis muscle; and, secondly, by puncture through the cartilage. The former method is that most generally adopted; but if the cyst be imperfect, as is often the case, or if adherent to the tarsal cartilage, as in the present instance, it could not be removed entire, and hence the cure would be incomplete without further treatment. In this case we shall therefore adopt the latter method, and rely upon gentle stimulation of the surface of the sac, after its contents are discharged, for the perfection of the cure.

The lid was now everted, and a small incision made with a scalpel, into the sac—by slight pressure a quantity of matter about the consistence of thick cream escaped, and the tumour subsided: a plug of lint was pushed through the opening into the sac, and allowed to remain. The patient presented herself at the next prescribing day; when a small piece of the sulph. cupri was introduced into the sac, and its surface lightly cauterized.

January 21. She again returned; the sides of the sac had closed, and the wound by which it was opened entirely healed up. Discharged cured.

The next case, to which we have to direct your notice, is that of Catharine Callahan, aged 37. Early in the summer of 1841, she unfortunately got a piece of quick lime into the right eye, which was of course followed by severe inflammation—which was treated, as she tells you, by the usual antiphlogistic remedies. In about six months the left eye became sore, and soon after this, or in December, 1841, she was admitted into the hospital by our colleague, Dr. Hays, for ophthalmia, ulcers, and opacity of the cornea, and came under our care when we took charge of the house, on the first of January, 1842. Under the usual treatment, she had so far improved by the April following, that her husband requested her discharge, although she was not well. Her sight remained pretty good for a month after she left, when a renewed inflammation of both eyes occurred, followed in a short time by such an opacity of the cornea, that she could not make her way about. In this condition she remained all summer until the 7th of September, when she again applied for admittance into the hospital. The improvement was then rapid for about three months, but since the middle of December it has been more slow. The sight of the right eye, which first became inflamed, is now the best—that of the left eye is very defective, the treatment not having had so beneficial an effect upon it as upon the other. When admitted in September, besides the opaque cornea, the upper lids of both eyes were covered with granulations. These, as you see, have been removed, and both lids now present very nearly a healthy appearance.

The opacity of the cornea has persisted for so long a period, that the ordinary remedies have ceased to exert a perceptible influence over it; or, at least, the improvement is so slow, as to induce us to make an effort to hasten it.

It is the result originally of an attack of conjunctivitis, in which the cornea has become involved secondarily.

The thickened state of the conjunctiva covering the globe of the eye is very evident, and you see a number of enlarged varicose vessels traversing it, and passing in upon the cornea. Now the object of our treatment is, to diminish the calibre of these vessels, and cut off the undue supply of blood which feeds this opaque portion of the cornea. This we can generally accomplish by astringent washes dropped upon the eye, and persevered in for a long time, or when the lids are granular, by the application of sulph. cupri to the granulations; but sometimes the structure of the conjunctiva has become so far altered, as to render this treatment ineffectual, without a resort to some other means.

A plan, which we have frequently adopted in this house, with marked advantage is, to clip out a small fold of the conjunctiva, in which one or more of these enlarged vessels are included, and thus to prevent the farther passage of blood in that direction. This is done by separating the lids, and requesting the patient to turn the eye in the desired direction, when the operator carefully seizes a fold of the conjunctiva with a small sharp hook made for the purpose, passes a pair of sharp curved eye-scissors behind it, and clips it off. A very small piece is generally sufficient, and this should be taken in most instances from the upper half of the eye, near the edge of the cornea, as the vessels are most numerous in this situation, originating, as they generally do, from the conjunctiva lining the upper lid. In performing this operation, care must be taken not to lacerate the membrane, or wound the sclerotic; nor should we attempt too much at once: it is better to clip out one piece at a time, and repeat the operation if necessary.

A small piece of the conjunctiva, just above the superior edge of the cornea, was now seized with the hook, and cut off—and the patient was advised to encourage the bleeding which followed by the application of warm water.

